# Tuméfaction cervicale chez une enfant au CHU de Brazzaville, République du Congo

**DOI:** 10.48327/mtsi.v3i1.2023.340

**Published:** 2023-03-14

**Authors:** Alexis Elira Dokékias, Firmine Olivia Galiba Atipo Tsiba, Fortuné Bissiko, Antoine Martin

**Affiliations:** 1 Service d'hématologie du CHU de Brazzaville et Centre national de référence de la drépanocytose (CNRD) « Antoinette Sassou N'Guesso », Brazzaville, République du Congo; 2 Service d'ORL du CHU de Brazzaville, République du Congo; 3 Département de cytologie et anatomie pathologiques, Hôpital Avicenne, Bobigny, France

**Keywords:** Tuméfaction cervicale, Enfant, Histiocytose sinusale, Maladie de Destombes-Rosaï-Dorfman, Corticoïdes, Brazzaville, République du Congo, Afrique subsaharienne, Cervical tumefaction, Child, Sinus histiocytosis, Destombes-Rosaï-Dorfman disease, Corticosteroids, Brazzaville, Republic of the Congo, Sub-Saharan Africa

## Abstract

Une écolière de 8 ans, originaire d'Afrique de l'Ouest, sans antécédents pathologiques, est admise en consultation d'hématologie au CHU de Brazzaville pour la prise en charge d'adénopathies cervicales. Le diagnostic d'histiocytose sinusale ou maladie de Destombes-Rosaï-Dorfman a été retenu et la patiente a été traitée par corticoïdes PO (méthylprednisolone 32 mg/j puis 16 mg/j). Compte tenu de sa rareté et de l’étiopathogénie incertaine de ce syndrome, le traitement est mal codifié. Il comporte la corticothérapie, des immunomodulateurs et parfois une chimiothérapie, une radiothérapie ou la chirurgie, indiquées en cas de manifestations cliniques de compressions d'organes locaux. La maladie peut spontanément régresser. Sa bénignité ne justifie pas un traitement systématique en l'absence de complications.

## Abstract (en)

A 8-year-old schoolgirl from West Africa with no previous pathological history was admitted to the haematology department of the Brazzaville University Hospital for the management of cervical adenopathy. The diagnosis of sinus histiocytosis or Destombes-Rosaï-Dorfman disease was retained and the patient was treated with PO corticosteroids (methylprednisolone 32 mg/d then 16 mg/d). Given the rarity and uncertain aetiopathogeny of this syndrome, treatment is poorly codified. It includes corticosteroid therapy, immunomodulators and sometimes chemotherapy, radiotherapy or surgery, indicated in case of clinical manifestations of local organ compression. The disease may regress spontaneously. Its benignity does not justify systematic treatment in the absence of complications.

Une écolière de 8 ans, originaire d'Afrique de l'Ouest, sans antécédents pathologiques, est admise en consultation d'hématologie au CHU de Brazzaville pour la prise en charge d'adénopathies cervicales. Elle se plaint depuis 3 mois d'une tuméfaction sous-mandibulaire et latéro-cervicale progressive apparue récemment, accompagnée d'une gêne à la déglutition, d'une dysphonie, d'une fièvre vespérale et de sudations abondantes. Elle a reçu avant l'admission un traitement oral par amoxicilline + acide clavulanique et ibuprofène sans amélioration des symptômes.

L'examen physique initial objective une obésité modérée et une bonne coloration des conjonctives. L'examen buccal est sans particularités. On observe des adénopathies sous-angulo-maxillaires et rétro-auriculaires bilatérales de 1 à 2 cm de diamètre, fermes, indolores et mobiles (Fig. 1). Il n'y a pas d'atteinte des nerfs crâniens, la nuque est souple, l'examen des oreilles par otoscopie est sans particularités de même que l'examen de la peau.

**Figure 1:**
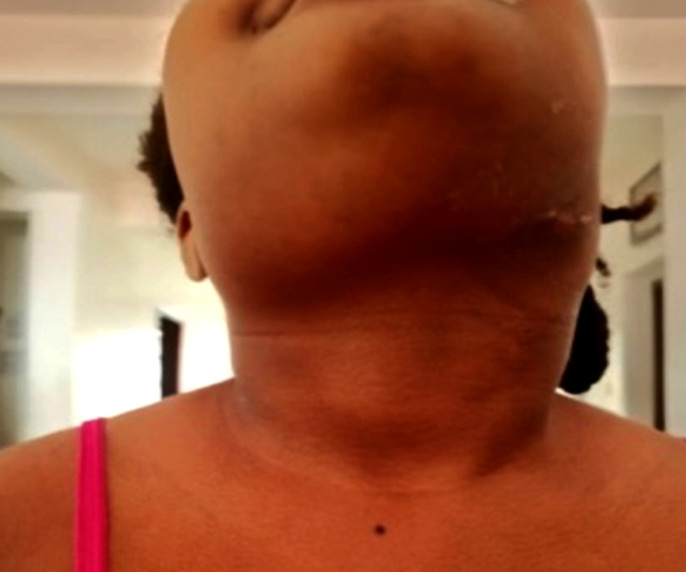
Présentation initiale Initial presentation

Les examens biologiques sont les suivants: leucocytes 8,45 G/l, hématies 4,210 G/l, hémoglobine 9,4 g/dL, hématocrite 28,2%, VGM 67 fl, CCMH 32 g/dl, plaquettes 596 G/l, VS 133 mm à la 1^re^ heure, LDH 238 U/L.

Plusieurs hypothèses sont évoquées dont une infection à virus Epstein Barr et la patiente reçoit avant le diagnostic final plusieurs traitements à visée antivirale ou immunomodulatrice (aciclovir, azithromycine, hydroxychloroquine).

L'examen anatomopathologique des biopsies ganglionnaires objective de volumineux ganglions de 3 × 2 × 1,10 cm, de consistance molle. L'histologie standard montre un aspect d'empéripolèse (Fig. 2) et une histiocytose sinusale majeure (Fig. 3). En immunohistochimie, la population histiocytaire sinusale est positive pour la PS100 (Fig. 4). Les colorations au PAS et de Ziehl sont négatives.

**Figure 2:**
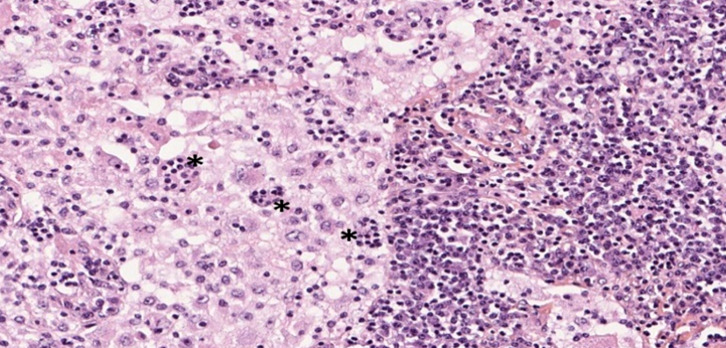
Aspect d'empéripolèse Aspect of emperipolesis

**Figure 3:**
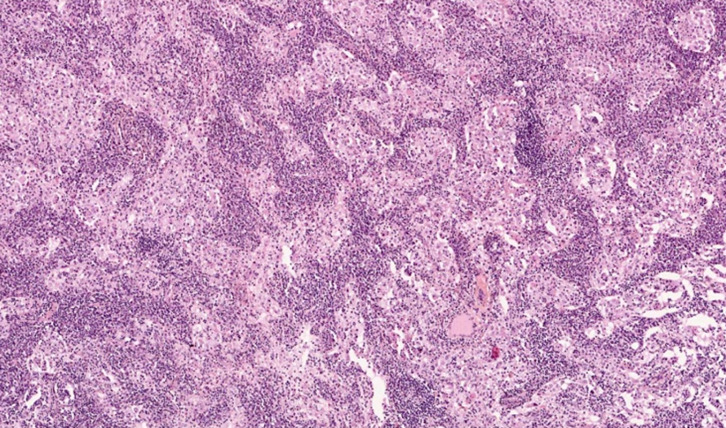
Histiocytose sinusale Sinus histiocytosis

**Figure 4:**
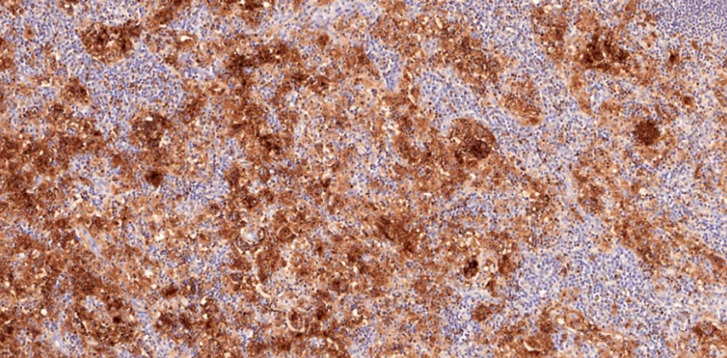
Immunohistochimie, positivité diffuse de la population histiocytaire sinusale pour la PS100 Immunohistochemistry, diffuse positivity of the sinus histiocyte population for PS100

Le diagnostic d'histiocytose sinusale ou maladie de Destombes-Rosaï-Dorfman est donc retenu et la patiente est traitée par corticoïdes PO (méthylprednisolone 32 mg/j puis 16 mg/j). Les adénopathies diminuent de taille dès J3 (Fig. 5). À J10, on note une régression sensible des adénopathies et la disparition des signes généraux.

**Figure 5:**
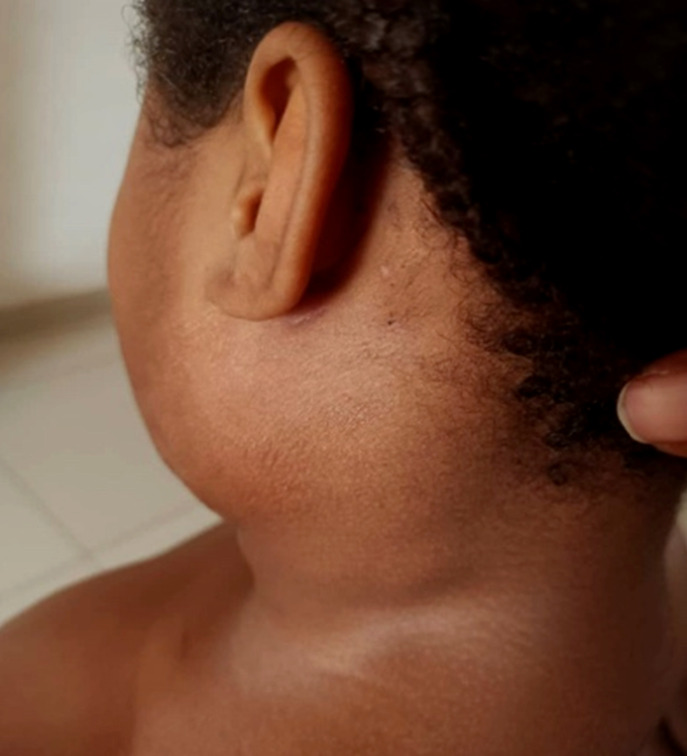
Aspect à J3 après traitement par corticoïdes Appearance at D3 after corticosteroid treatment

La maladie de Destombes-Rosaï-Dorfman est une histiocytose non langerhansienne caractérisée classiquement par une atteinte ganglionnaire cervicale massive, localisée. Plus rarement, on observe des formes atypiques ou disséminées, cutanées, naso-sinusiennes, orbitaires ou osseuses. L'histiocytose sinusale est rare et cosmopolite. Des cas, surtout ganglionnaires chez des enfants, ont été rapportés en Afrique subsaharienne, posant des problèmes de diagnostic clinique avec des adénopathies virales (mononucléose, toxoplasmose, VIH), bactériennes (tuberculose, histoplasmose) ou tumorales (lymphomes, Burkitt) [2, 3, 4, 5].

Sur le plan étiopathogénique, aucun travail scientifique n'a permis de conclusion formelle quant à l’étiologie de ce syndrome. L'expression de la pathologie concerne l'activation des phagocytes mononucléés. Aucune cause infectieuse n'est identifiée à ce jour, mais le rôle d'un virus de la famille des Herpes comme le virus Epstein Barr n'est pas à exclure [1].

La maladie touche principalement les enfants et les adultes jeunes. Le signe clinique le plus fréquent est la présence d'adénopathies cervicales volumineuses (90% des cas) [1]. Un syndrome inflammatoire biologique, parfois accompagné d'une anémie inflammatoire est fréquent. L’électrophorèse des protéines sériques révèle dans près de 70% des cas une hypergammaglobulinémie polyclonale.

Le diagnostic positif est histologique et posé devant:

- une accumulation d'histiocytes à noyaux ronds, de grande taille, fortement nucléolés;
- un large cytoplasme éosinophile de ces cellules, parfois xanthomisé (cellules de Destombes), qui sont constamment CD68 positives, CD1a négatives et le plus souvent DS100 positives (Fig. 4);
- la présence des lésions d'empéripolèse, non spécifiques.

Compte tenu de sa rareté et de l’étiopathogénie incertaine de ce syndrome, le traitement est mal codifié. Il comporte la corticothérapie, des immunomodulateurs et parfois une chimiothérapie, une radiothérapie ou la chirurgie, indiquées en cas de manifestations cliniques de compressions d'organes locaux. La maladie peut spontanément régresser. Sa bénignité ne justifie pas un traitement systématique en l'absence de complications.

## Liens D'intérêts

Les auteurs ne déclarent aucun lien d'intérêt.
